# Effect of gallic acid on electrophysiological properties and ventricular arrhythmia following chemical-induced arrhythmia in rat

**DOI:** 10.22038/IJBMS.2019.33296.7948

**Published:** 2020-02

**Authors:** Ghaidafeh Akbari, Mahin Dianat, Mohammad Badavi

**Affiliations:** 1 Department of Physiology, Persian Gulf Physiology Research Center, Faculty of Medicine, Ahvaz Jundishapur University of Medical Sciences, Ahvaz, Iran

**Keywords:** Arrhythmia, Electrophysiological – properties, Gallic acid, Ventricular, Rat

## Abstract

**Objective(s):**

Ventricular arrhythmias including ventricular tachycardia (VT) and ventricular fibrillation (VF) are the most important causes of mortality rate. Gallic acid (GA) has beneficial effects on cardiovascular diseases. The aim of this study was to evaluate the effects of GA on electrophysiological parameters such as QRS complex, heart rate (HR), PR interval parameters, and ventricular arrhythmia following chemical induction in rat.

**Materials and Methods:**

Seventy-two male rats were divided into 9 groups (n=8). Chronic groups pretreated by GA (10, 30, and 50 mg/kg, orally) and normal saline (N/S, 1 ml/kg, orally) for 10 days. At the start of the experiments (the first day) and on the final day of the experiments (tenth day), the electrocardiogram (lead II) was recorded. At acute group, GA (50 mg/kg), and anti-arrhythmic drugs such as propranolol, amiodarone, and verapamil injected via intravenous (IV). Then, arrhythmia induced by a CaCl_2_ 2.5% solution (140 mg/kg, IV). Afterward, percentage of premature ventricular beats (PVB), VF, and VT were recorded at 1, and 3 min.

**Results:**

These findings showed that chronic and acute doses of GA have positive inotropic and anti-dysrhythmic effects by significant reduction of PVB, VT and VF on comparison with the control group. These actions are comparable to anti-arrhythmic drugs such as quinidine, propranolol, amiodarone, and verapamil. GA has not significant effect on chronotropic and dromotropic properties.

**Conclusion:**

Findings showed that GA has antiarrhythmic, and inotropic characteristics that suggested GA has effective for mild congestive heart failure, and cardiovascular disorders patients which susceptible to incidence of arrhythmias.

## Introduction

Cardiovascular diseases (CVDs) are the first reason of death in worldwide. CVDs were occurred in several conditions such as heart failure, coronary artery disease, and arrhythmia (1). Arrhythmia resulted from irregularly at impulse generation, impulse conduction in cardiac or combination of both (2). The most important etiologies of cardiac arrhythmias are congenital heart diseases, myocardial ischemia, cardiac valvular diseases, electrolyte imbalances, metabolic disturbances, acidosis or alkalosis, and drugs toxicity (3).

The different types of ventricular arrhythmia are including premature ventricular beat (PVB), ventricular tachycardia (VT), and ventricular fibrillation (VF). VT, and VF are the most important causes of mortality rate (4). Fibrillation is an arrhythmia which describe by irregular rhythm and VT is an arrhythmia with further than 4 PVB/minute (5). 

Anti-arrhythmic drugs which administered for treatment or protection against arrhythmia, have many side effects which limits using of them (6)**.** These drugs are divided into four major groups; based on Williams Wagon classification; such as sodium channel blockers (quinidine) (7), beta blocker drugs (propranolol) (8), potassium channel blockers (amiodarone) (9), and calcium channel blockers (verapamil) (8). 

With regard to efficacy and safety, natural materials are preferred to synthetic products (10). Because reduced costs and complications, the use of medicinal plants are preferred to chemical drugs (11). The beneficial effects of plants may be attributed to the presence of anthocyanins, ﬂavonoids, and other phenolic compounds (12). In this regard, gallic acid (GA) with its anti-oxidant actions and potent free radical scavenging received much attention (13).

GA is a potent anti-oxidant belongs to the larger group of plant polyphenols known as gallotannins. It founded in vegetables, fruits (14), tea leaves, grapes, blackberry, and gallnuts (15). It has multiple biological effects including anti-allergic, anti-microbial, anti-cancer, anti-ulcer, anti-hyperglycemic, lipid homeostasis. and neuroprotective (16-22). It reduces cardiac troponin-T, lactate dehydrogenase, and creatine kinase in serum (23). 

Many evidences reported that GA is a promising CVDs agent (24-26). Therefore, we evaluate the effect of GA pretreatment on electrophysiological parameters such as inotropic, chronotropic, dromotropic and ventricular arrhythmia are including VF, VT, and, PVB following chemical-induced arrhythmia in rat. 

## Materials and Methods

***Animals***

Seventy-two adult male Sprague Dawley Rats (200-250 g) were prepared from the animal house of Ahvaz Jundishapur University of Medical Sciences, Ahvaz, Iran. Rats were fed on a conventional diets and tap water *ad libitum*. They were maintained under standard conditions of humidity (50%), temperature (20-24 °C), and 12 hr light–dark cycle. At this duration time, gavage was done with GA solution by oral-gastric tube. Animals were deprived of food, but not water, overnight before experiments. All experiments were performed in accordance with Ethics Committee of Ahvaz Jundishapur University of Medical Sciences.

***Preparation of animals***

First, animals were subjected to anesthesia with intra peritoneal (IP) injection of 50 mg/kg ketamine and 10 mg/kg xylazine. The femoral vein was cannulated for drug injection. The electrocardiogram (ECG) changes were monitored via lead II of ECG by Bio-Amp and power lab set up (ADInstruments Co., Australia). Interpretation of electrophysiological parameters was based on computer analyses of ECG. 

***Animal grouping***

Seventy-two rats were randomly divided into 9 groups (eight to each):

Group I: received normal saline (N/S), 1 ml/kg, 10 days, orally. 

Group II: pretreated by GA, 10 mg/kg, 10 consecutive days, orally. 

Group III: pretreated by GA, 30 mg/kg, 10 consecutive days, orally.

Group IV: pretreated by GA: 50 mg/kg, 10 consecutive days, orally.

Group V: pretreated by GA, 50 mg/kg, IV slowly. 

Group VI: pretreated by quinidine, 5 mg/kg, IV slowly.

Group VII: pretreated by propranolol, 2 mg/kg, IV slowly.

Group VIII: pretreated by amiodarone, 10 mg/kg, IV slowly. 

Group IX: pretreated by verapamil, 5 mg/kg, IV slowly.

***The route of recording of electrophysiological parameters and CaCl***_2–_ ***induced arrhythmias***

At this procedure, chemical-induced arrhythmia was done by CaCl_2_ (140 mg/kg, IV). First, at all groups 15 min post**-**operation, heart rate (HR), QRS complex and PR interval were calculated by lead II. Then, CaCl_2_ injected, incidence of PVB, VT, and VF were calculated at 1, and, 3 min as percentage.

***Statistical analysis***

Results were shown as Mean±SEM. Comparisons among groups were represented using one-way ANOVA, FISHER exact test, or t-test. *P*<0.05 were considered significant. 

## Results

As illustrated in Table 1, QRS complex was significantly decreased following chemical-induced arrhythmia**. **The doses of 30 and 50 mg/kg of GA were shown a significant effect on amplitude of QRS complex in comparison with control. The data demonstrateed that GA has no significant effect on HR and PR interval.

***The effect of chronic and acute doses of GA on CaCl***_2_ ***-induced arrhythmia in comparison with the quinidine***

**Figure 1:**
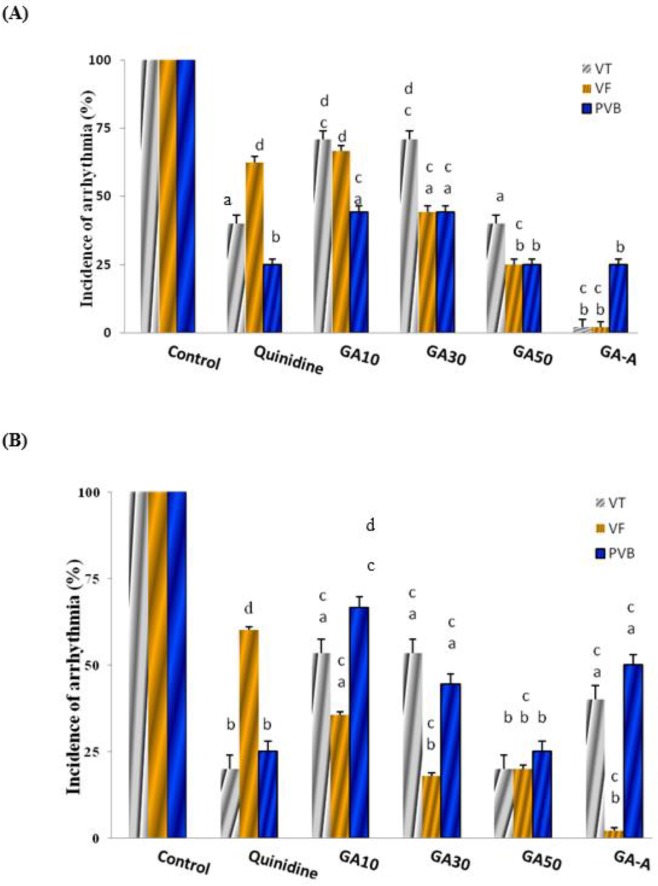
The effect of chronic (10, 30, 50 mg/kg) and acute (50 mg/kg) doses of GA on CaCl_2_-induced arrhythmia after 1 (A) and 3 (B) min in comparison with quinidine (5 mg/kg). Data in control group was considered as 100% and were compared to those of other groups. GA: Gallic acid; A: Acute; PVB: Premature ventricular beats; VF: Ventricular fibrillation; VT: Ventricular tachycardia. d=*P*<0.05, a=*P*<0.01, b=*P*<0.001 vs. control, c=difference with the quinidine

As showed in Figure 1 (A and B) the antiarrhythmic effects of 10, 30, 50 mg/kg GA (chronic doses) and acute (GA-A, 50 mg/kg) indicated that incidence of PVB, VT, and VF at 1 min significantly reduced in dose dependent manner in compared to the control group (*P*<0.05). GA-A had better effect than other groups (*P*<0.001). Quinidine and chronic GA at dose of 10 mg/kg had equal and less effect than other groups respectively.

***The effect of acute and chronic doses of GA on CaCl***_2_ ***-induced arrhythmia in comparison with the propranolol***

**Figure 2:**
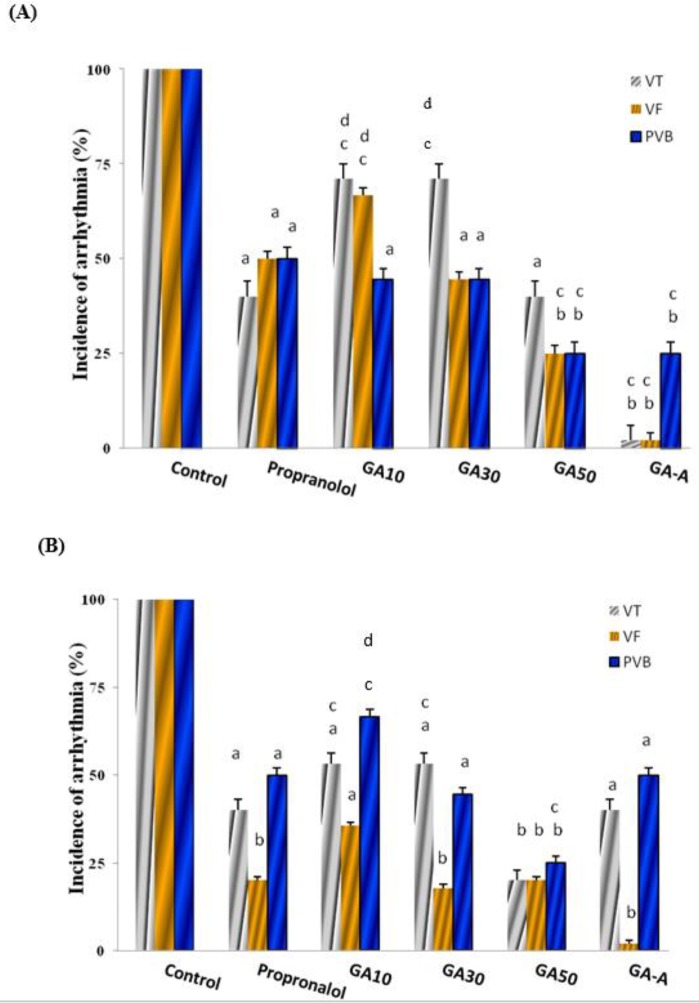
The effect of chronic (10, 30, 50 mg/kg) and acute (50 mg/kg) doses of GA on CaCl_2_-induced arrhythmia, after 1 (A) and 3 (B) min in comparison with propranolol (2 mg/kg). Data in control group was considered as 100% and were compared to those of other groups. GA: Gallic Acid; A: Acute; PVB: Premature ventricular beats; VF: Ventricular fibrillation; VT: Ventricular tachycardia. d=*P*<0.05, a=*P*<0.01, b=*P*<0.001 vs. control, c=difference with the propranolol

The results showed that GA at chronic (10, 30, 50 mg/kg) and acute (GA-A, 50 mg/kg) doses had significant reduction on incidence of PVB, VT and VF at first and 3 min compared to control group (*P*<0.05), in a dose dependent manner. The GA-A had better effect than other groups (*P*<0.001) at 1 min. In comparison among groups, GA (10 mg/kg) had equal effect with propranolol (Figures 2-A and B). 

***The effect of acute and chronic doses of GA on CaCl***_2_ ***-induced arrhythmia in comparison with the amiodarone***

**Figure 3:**
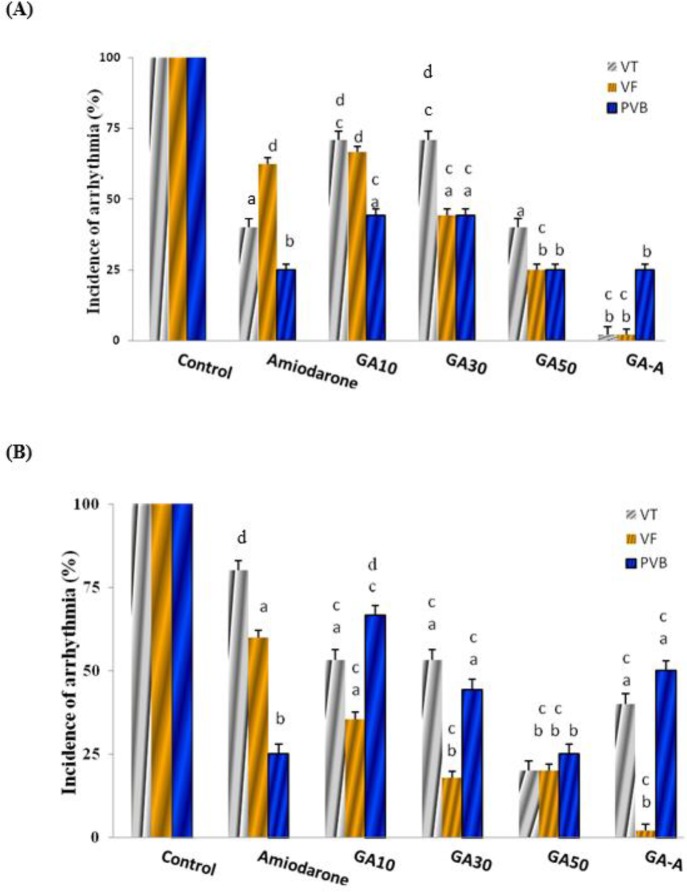
The effect of chronic (10, 30, 50 mg/kg) and acute (50 mg/kg) doses of GA on CaCl_2_-induced arrhythmia after 1 (A) and 3 (B) min in comparison with amiodarone (10 mg/kg). Data in control group was considered as 100% and were compared to those of other groups. GA: Gallic Acid; A: Acute; PVB: Premature ventricular beats; VF: Ventricular fibrillation; VT: Ventricular tachycardia. d=*P*<0.05, a=*P*<0.01, b=*P*<0.001 vs. control, c=difference with the amiodarone

The results showed that GA at chronic (10, 30, 50 mg/kg) and acute (GA-A, 50 mg/kg) doses had significant reduction on incidence of PVB, VT, and VF at 1and 3 min compared to control (*P*<0.05), in dose dependent manner. It was showed that GA-A had better effect than other groups (*P*<0.001) at 1 min (Figures 3-A and B). In comparison among groups, GA (10 mg/kg) had equal effect with amiodarone. 

***The effect of acute and chronic doses of GA on CaCl***_2_ ***-induced arrhythmia in comparison with the verapamil***

**Figure 4:**
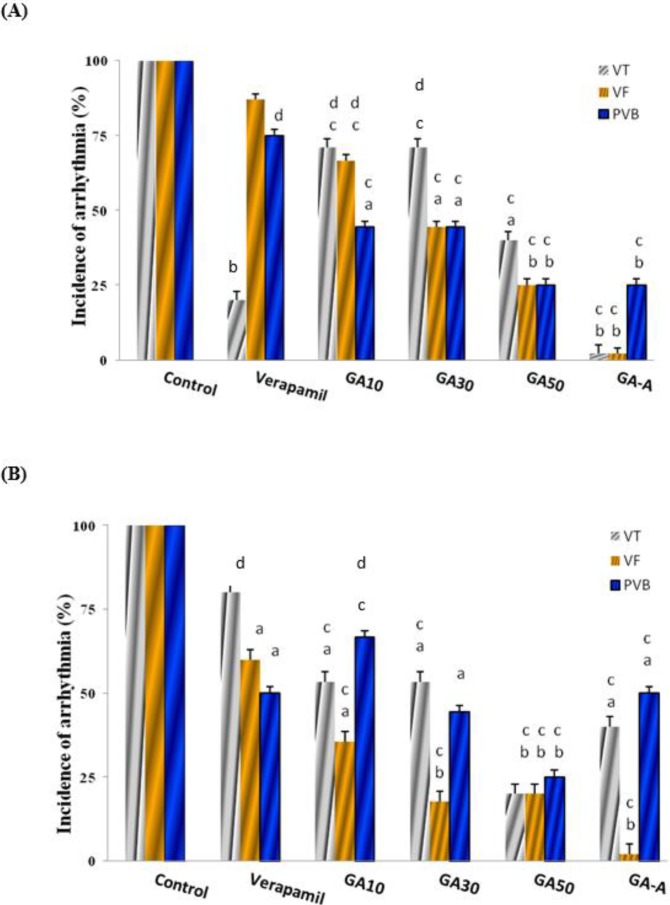
The effect of chronic (10, 30, 50 mg/kg) and acute (50 mg/kg) doses of GA on CaCl_2_-induced arrhythmia after 1 (A) and 3 (B) min in comparison with verapamil (2 mg/kg). Data in control group was considered as 100% and were compared to those of other groups. GA: Gallic acid; A: Acute; PVB: Premature ventricular beats; VF: Ventricular fibrillation; VT: Ventricular tachycardia. d=*P*<0.05, a=*P*<0.01, b=*P*<0.001 vs. control, c=difference with the verapamil

As showed in Figure 4 (A and B), the results showed that the 10, 30, 50 mg/kg of GA (chronic doses) and acute (GA-A, 50 mg/kg) had significant reduction on incidence of PVB, VT and VF at 1 and 3 min in compared to the control group (all *P*<0.05), in a dose dependent manner. GA-A had better effect than other groups (*P*<0.001) at 1 min. In comparison among groups GA (10 mg/kg) had equal effect with verapamil, and other groups had further effect.

**Table 1:** The effect of chronic doses of gallic acid on electrophysiological parameters in different groups

**Electrophysiological parameters**						
**PR Interval** ** (msec)**		**QRS Complex** ** (mv)**		**HR** ** (bpm)**		
**After**	**Before**	**After**	**Before**	**After**	**Before**	**Groups**
0.075±0.003	0.07±0.004	0.21±0.01	0.19±0.01	271±7	273±8	**Control** **(N/S, 1 ml/kg)**
0.075±0.005	0.07±0.003	0.21±0.012	0.19±0.01	277.5±7	275±7	**GA (10 mg/kg)**
0.085±0.005	0.073±0.004	0.23±0.015*	0.16±0.011	266±11	285±7	**GA (30 mg/kg)**
0.074±0.006	0.062±0.007	0.24±0.015*	0.16±0.012	273.8±8	275.6±11	**GA (50 mg/kg)**

## Discussion

This study showed that GA has anti-dysrhythmic effects on CaCl_2_-induced arrhythmias by decreasing the incidince of VF, VT, and PVB in comparison with anti-arythmic drugs. GA also had positive inotropic action in comparison with rats in the control group. 

The previous study, demonsterated that galate compounds attach to lipid membrane, and operated as anti-oxidant factor. These agents remarkably decreases levels of cadiac marker enzymes that disturbance structural, and functional of cardiac, and increase non-enzymatic activity of anti-oxidants in plasma such as glutathion, vitamins E and C (23). Based on the observations presented here, GA has protective effects on cardiac dysrhythmia by decreasing the incidince of VF, VT, PVB and increasing QRS complex amplitude as positive inotropic effect. In agreement, it has been shown that GA inhibits oxidant agents and eventually increase QRS complex voltage which lead to positive inotropic effect (27).

It has been reported that increased risk of ventricular arrhythmias can be accompany with prolongation of QT interval (28) which pretreatment with GA increased QRS voltage, and reduction of QT interval in rat model of liver cirrhosis following induction of bile duct ligation (29). In agreement, another study showed that pretreatment with GA eliminated doxorubicin-induced alterations ECG such as prolongation of QT interval, ST segment, QRS complex, and P wave reduction (30).

GA as an anti-oxidant element can be used for cardiovascular protection against thrombosis due to inhibition of platelet-leukocyte interaction, P-selectin expression , and platelet aggregation (31). Furthermore, one study presented that GA preserves the lysosomal membrane damage in cardiac injury model induced by isoproterenol, and return the activities of lysosomal enzymes to near normal levels (27). Indeed, it is indicated that GA, and linoleic acid esters may act as a strong hypolipidemic agent against triglyceride, as well as low-density lipoprotein- cholesterol (LDL-c) (32). Polyphenol of GA that exists in *Vitis vinifera *(grape seed) has anti-oxidant effect 50 folds vitamin C and 20 folds vitamin E which decline ionized calcium intra cellular (33, 34). Therfore, GA as an important lipid profile decreasing agent may be protect cardiovascular problems following hyperlipidemia (35).

Studies showed that GA through increasing the capacity of endogenous anti-oxidant system protected the rat isolated heart against Ischemia Reperfusion (IR)-induced injury (36, 37). Reperfusion-induced arrhythmias are the most common etiologies of sudden cardiac death, and can be generated in humans and experimental animals. VT, VF, and PVB are the most important arrhythmias which happens due to calcium excess during early minutes of reperfusion and overproduction of ROS (38). 

These findings of our study suggest that GA similar to methyl maslinate, ursolic acid uvaol, and oleanolic acid has cardiotonic and anti-dysrhythmic effects following CaCl_2_-induced arrhythmias (39). 

It has been shown that pretreatment with propranolol prevented the increase cAMP/PKA in acute phase of myocardial infarction (MI). Since, GA modulated of cAMP, and intracellular calcium, therefore, may be has common mechanism in anti-arrhythmic against chemical-induced arrhythmias (40). Propranolol has a membrane stabilizing effects because inhibition of sodium channels. This property may be explaining some effective role of propranolol in remedy of arrhythmias (27, 41). Furthermore, it has some clinical benefit actions and is used to treat cardiovascular diseases by stabilizing the inactivated of the channel (42).

Significant characteristic of amiodarone is prolong action potential due to inhibition of i_k_ channels and, then increase effective refractory period that decrease capability of cardiac for accelerared tachycardia (43). Amiodarone also terminated arrhythmias via inhibition of sodium and, calcium channels. The effect of amiodarone on electrophysiology properties is prolongation of PR interval (9). Therfore, we speculate that GA probably through similar mechanisms inhibits these arrhythmias.

## Conclusion

The results of this study such as increase in QRS complex voltage, and decrease in PVB, VT, and VF in GA pretreatment groups demonstrated inotropic, and anti-arrhythmic properties of GA as a protective agent in heart diseases. This effect of GA is comparable with anti-arrhythmic drugs. Therfore, it can be suggested that GA will be effective for cardiovascular disorders persons, mild chronic heart failure, and patients in the face of multiple interventions which are susceptible to incidence of arrhythmias in the heart. 
